# Gender identity, sexual orientation and adverse sexual experiences in autistic females

**DOI:** 10.1186/s13229-020-00363-0

**Published:** 2020-07-11

**Authors:** Laura A. Pecora, Grace I. Hancock, Merrilyn Hooley, David H. Demmer, Tony Attwood, Gary B. Mesibov, Mark A. Stokes

**Affiliations:** 1 Department of Psychology, School of Psychology, Faculty of Health, Deakin University, 221 Burwood Hwy, Burwood, Victoria 3125 Australia; 2 Specialist Clinic for Autism Spectrum Conditions, Minds & Hearts: A Clinic for Autism Spectrum Conditions, Brisbane, Australia; 3 Division TEACCH, University of North Carolina, Chapel Hill, NC USA

**Keywords:** Autism, Autism spectrum disorder, Female sexuality, Gender identity, Sexual orientation, Sexual vulnerabilities

## Abstract

**Background:**

There is growing recognition that autistic females present with more diverse gender and sexual identities than their non-autistic counterparts. Likewise, autistic females are also at an increased risk of adverse sexual experiences. As higher rates of sexual victimisation are observed in individuals with diverse sexual identities in the broader population, rates of negative sexual experiences among autistic females remain unclear. This study aimed to investigate the representation of gender and sexual diversity within autistic females and examine their rates of regretted, and unwanted, sexual encounters among females with a transgender gender identity and non-heterosexual sexual orientation.

**Methods:**

Two hundred and ninety-five females completed the Sexual Behaviour Scale-III (SBS-III) online. Self-reported gender identity and sexual orientation were compared between 134 autistic (*M*_age_*=* 26.2 years, SD *=* 8.7) and 161 non-autistic females (*M*_age_ = 22.0 years, SD = 4.6). Differences in the prevalence of negative sexual experiences were compared across diagnosis and each gender identity and sexual orientation label.

**Results:**

Autistic females were more likely to identify with a transgender gender identity (*p* < .05) and non-heterosexual sexual orientation (*p* < .007) compared to non-autistic females. Autistic homosexual females were more likely to have experienced a range of negative sexual experiences than autistic heterosexual females (OR ≥ 3.29; *p* < .01) and were more likely to have experienced unwanted sexual experiences than non-autistic females regardless of sexual orientation (OR ≥ 2.38; *p* < .05). There were no differences in rates of negative sexual experiences between autistic bisexual and both autistic heterosexual and non-autistic bisexual females. Non-autistic bisexual females (OR *=* 0.24; *p* = .018) presented with a reduced risk of regretted sexual experiences than non-autistic heterosexual peers. There were no differences in negative sexual experiences across gender identity in the autistic sample.

**Limitations:**

The use of fixed format response items may have restricted participants’ abilities to provide rich responses pertaining to their sexual identities and nature of negative sexual experiences. The small number of participants who identified as transgender (*n* = 40) limits the reliability of results pertaining to sexual experiences across gender identity. Moreover, although multiple recruitment methods were used in this study, non-representative may bias estimates of prevalence rates. Thus, the data may not be representative of the broader population.

**Conclusions:**

Results indicate that autistic females present with greater diversity in their sexual identities than individuals without autism, with those with a homosexual sexual orientation being at greater risk of experiencing adverse sexual encounters. Findings suggest the importance of increased clinical attention to this diversity and the need to provide support to facilitate the development of a healthy sexual identity and reduce the risks identified in this study.

## Background

Within autism, there appears to be greater variability in gender identity than found in the wider population. Studies proposing this link have identified an increased prevalence of gender variance in autism. Defined as an umbrella term that describes variability between an individual’s assigned sex and gender identity [1, 2], gender variance has been reported by 5.4 to 7.2% of autistic natal male and female youths [1, 2] and 11.3% of autistic adults [3], yet between 0.7 [1] and 5% [3] in non-autistic or control samples. A relationship between gender dysphoria (GD) and autism has also been suggested, where both a higher representation of GD in autistic groups irrespective of sex, as well as an increased number of autistic symptoms in adults with GD, are common observations within current literature [3–5]. Sex differences have also been observed, where the co-occurrence of elevated rates of gender variance in autistic groups [6], as well as greater clinically significant autism symptoms in adult GD samples [7–9], is reported in individuals assigned female at birth than individuals assigned as male. Despite this, studies investigating the relationship between autistic traits and GD in child samples have found comparable levels of autism symptoms between boys and girls with GD [10, 11].

According to the American Psychological Association [12], gender identity is defined as an individuals’ internal conception of themselves as male, female, a blend of both, or otherwise. As gender identity is self-identified, it is often informed by an individual’s personal perceptions and experiences of their own masculinity or femininity [13]. Gender identity is also intimately linked with gender roles: the behaviours and attitudes a given society considers acceptable and appropriate for individuals based on their sex as male or female [12, 14]. Consequentially, there are often notable variations in the attitudes and behaviours perceived as typical to each gender across cultures [6]. For the purpose of this study, all individuals assigned a female sex at birth are referred to as female. Throughout, the term ‘(autistic/non-autistic) female’ has been used in relation to all participants who reported being assigned female at birth. The term cisgender is used to describe gender identities that correspond with one’s birth sex. Thus, the term ‘cisgender female’ refers to an individual assigned female at birth, who self-identifies with a female gender identity. Transgender is used to describe gender identities that are inconsistent with birth sex. In these instances, ‘transgender female’ is used to describe an individual who identifies with a non-female gender identity. The term ‘female’ refers to an individual assigned as female at birth, irrespective of gender identity. Care and consideration have been taken to consider appropriate language when referencing gender identity and sexual orientation.

Another common observation within the sexuality literature is an increased rate of non-heterosexual sexual orientations within the autistic population. Sexual orientation is defined as a multidimensional construct that largely includes three domains: sexual identity, sexual attraction, and sexual contact [14]. Each dimension exists along a continuum and reflects an individual’s underlying sexual preference towards others [15]. The terms ‘heterosexual’ and ‘homosexual’ are the most frequently used labels to describe sexual orientation [16, 17]. However, there are a range of alternative labels an individual may also identify with, including, ‘lesbian’, ‘gay’, and ‘bisexual’ [17]. When compared to non-autistic groups, studies have observed greater identification with non-heterosexual sexual orientations [14, 18], as well as higher rates of non-heterosexual interests, among those with autism [19]. Autistic females also present with greater variability in their sexual orientation. This has been evidenced by higher rates of bisexual and lesbian sexual orientations, as well as lower rates of heterosexuality in autistic females when compared to both autistic males [4, 6, 20], and non-autistic females [21]. Within this study, the term non-heterosexual/homosexual sexual orientation is used to describe identification with lesbian or bisexual sexual orientation.

In addition to an increased diversity in both gender identity and sexual orientation, recent insights into the sexuality of autistic individuals have begun to identify a number of challenges and adverse experiences across a range of sexual and relationship-based domains [4, 6, 14, 22]. Some of these include reduced levels of sexual and romantic functioning, marked by lower engagement in sexual behaviours despite an expressed interest in sexuality and relationships [23, 24], increased difficulties maintaining relationships [19], and increased risks to sexual victimisation [25] compared to non-autistic peers.

An additional factor shaping the sexuality of autistic people is the nature of sexual experiences, and the increased sexual risks and vulnerabilities that have been identified among autistic females. Mediated by a lack of sexual knowledge [25], and difficulties facilitating social interactions [26], more autistic individuals are subject to a range of adverse sexual experiences including victimisation and abuse [25, 26]. Although some research has demonstrated that these issues appear to affect autistic individuals irrespective of sex [27], recent insights have identified that autistic females are subject to negative experiences at a greater rate than both male counterparts and non-autistic peers. For example, autistic females express less sexual interest, greater likelihood of engaging in sexual behaviours that are later regretted, and higher risk of being subject to unwanted sexual advances by others compared to autistic males [28]. Compared to non-autistic females, autistic females are more likely to have experienced sexual victimisation [17], unwanted sexual contact, and sexual coercion [25]. Similar observations have been reported within qualitative literature, which identified a number of striking themes within autistic female groups, including experiences of sexual victimisation due to being overly trusting [29], or misinterpreting the intentions of others [29]. Evidence of social naivety in autistic females impacts relationship experiences [30], as some females can be vulnerable to promiscuity when initiating desired relationships [31]. When taken together, the findings suggest that autistic females present with greater variations in gender identity and sexual orientation than both autistic males and the general population, as well as increased risks and vulnerabilities across a range of sex-related domains.

There is a substantial body of research that indicates that individuals identifying with transgender gender identities and non-heterosexual sexual orientations experience forms of sexual victimisation at higher rates than the general population [31–33]. Within all reviewed studies the prevalence of sexual assault [33], intimate partner violence, and victimisation is greatest among lesbian and bisexual females when compared to heterosexual counterparts [34]. In studies examining these variables across gender identity, transgender females report higher rates of sexual assault victimisation [35] and intimate partner violence [36] than cisgender comparisons. These individuals also often experience a range of poor physical and psychological health outcomes, including higher levels of depressive symptoms, substance abuse, and suicide risk [37, 38].

To date, links between adverse sexual experiences and higher incidences of both psychological distress and psychopathology have been identified within the broader population [39, 40]. However, research has not investigated the possibility of this relationship also occurring within autism, as well as within transgender and non-heterosexual groups. If a similar association is also found in the autistic transgender or autistic non-heterosexual population, the psychological outcomes that follow negative sexual events may further impact the existing challenges of poor psychological and subjective wellbeing and increased rates of comorbid mental health conditions, that are already evident within autistic [41, 42], transgender [37, 38], and non-heterosexual populations [43, 44].

Together, evidence suggests that females, those identifying as transgender and/or non-heterosexual might be at higher risk of experiencing negative sexual experiences than individuals within each respective population alone. However, there are currently no existing studies that have investigated rates of adverse sexual experiences across gender identity and sexual orientation within autism. As such, our study was undertaken to determine whether rates of negative sexual experiences are significantly greater for autistic females within these gender and sexual minority groups compared to non-autistic peers.

To date, the constructs of regretted, unwanted sexual experiences, and unwanted sexual advances have not been examined within autism. These three constructs have also not been examined together in research investigating sexuality within the broader population. Thus, test items used to measure these experiences were developed from the broader sexuality literature defining and describing rates of a range of adverse sexual experiences in the broader population [45–48]. Commonly cited experiences included engaging in sexual behaviours that were followed by feelings of regret [45]; engagement in unwanted sexual experiences, often in the form of sexual coercion and sexual victimisation [46]; and experiences of unwanted sexual advances [47].

The purpose of the current study was to examine differences between autistic females and their non-autistic female peers in (i) the representation of variations in gender identities and sexual orientations and (ii) the rate of negative sexual experiences for autistic and non-autistic females within these minority groups. In order to address this broader aim, three specific aims and hypotheses were proposed:

- *Aim 1:* To examine gender identity and sexual orientation in autistic females to determine if a greater proportion of autistic females identify as transgender or with a non-heterosexual sexual orientation than non-autistic females.
- *Hypothesis 1*. Compared to non-autistic females, a greater proportion of autistic females would identify as transgender or with a non-heterosexual sexual orientation.
- *Aim 2*. To determine if autistic transgender females are more likely to have experienced a negative sexual encounter than (i) autistic cisgender females and/or (ii) non-autistic cisgender or transgender females
- *Hypothesis 2*. Autistic transgender females (identifying as male or other) would be more likely to have engaged in a sexual behaviour that was regretted, unwanted, or report experiences of unwanted sexual advances than autistic cisgender females and non-autistic cisgender, or transgender females.
- *Aim 3*. To determine if autistic females identifying with a non-heterosexual sexual orientation are more likely to have experienced a negative sexual encounter than (i) autistic females identifying as heterosexual and/or (ii) non-autistic females identifying with a heterosexual, and non-heterosexual, sexual orientation
- *Hypothesis 3*. Autistic females identifying with a non-heterosexual sexual orientation (homosexual or bisexual) would be more likely to have engaged in a sexual behaviour that was regretted, unwanted, or report experiences of unwanted sexual advances than both autistic females identifying as heterosexual and non-autistic females identifying with a heterosexual and non-heterosexual sexual orientation.

## Method

### Participants

A total of 295 females (> 18 years of age) participated in this study. Of these, 161 were individuals without autism, whereas the remaining 134 self-reported a formal diagnosis of autism spectrum disorder (ASD [49];), confirmed by an Autism Quotient (AQ [50];) score above 32 points. Table 1 presents characteristics of the sample.

**Table 1:** Characteristics of study sample

Characteristics	Autistic, *n =* 134	Non-autistic, *n =* 161	*Z*_Diff_	*p* value
	*n* (%)	*n* (%)		
**Age range**	18-56 years	18-48 years		
**Mean age (SD)**	26.2 years (8.7)	22.0 years (4.6)		
**Comorbid diagnosis**				
ADHD	23 (17.2)	3 (1.9)	4.53	.001
Anxiety disorders^a^	23 (17.2)	18 (11.2)	1.49	.137
Bipolar disorder	2 (1.6)	2 (1.2)	0.29	.382
Borderline personality disorder	1 (0.7)	2 (1.2)	0.43	.363
Bulimia	0 (0.0)	1 (0.6)	2.25	.057
Depressive disorders^b^	38 (28.4)	18 (11.2)	3.73	.001
Epilepsy	0 (0.0)	1 (0.6)	2.25	.057
Non-verbal learning disorder	1 (0.7)	0 (0.0)	0.84	.307
Obsessive compulsive disorder	3 (2.2)	2 (1.2)	0.67	.320
Post-traumatic stress disorder	3 (2.2)	1 (0.6)	1.10	.231
Schizophrenia	1 (0.7)	0 (0.0)	0.84	.307
Tourette’s syndrome	1 (0.7)	0 (0.0)	0.84	.307
None/not reported	38 (28.4)	113 (70.3)	7.18	< .001

^a^Includes generalised anxiety disorder, social phobia, and panic disorder

^b^Includes major depressive disorder, persistent depressive disorder

Proportion tests (*Z* difference) conducted to examine differences in comorbid diagnosis between autistic versus non-autistic sample

Following screening of AQ scores, 11 autistic participants, who scored below the specified cut-off of 32, were excluded from further analysis. Analysis of the remaining sample identified significant group differences in mean AQ scores between the diagnostic groups with *M*_autistic_ higher (*M* = 39.94; SD = 5.93) than *M*_non-autistic_ (*M* = 24.24; SD = 11.29; *t*_(282)_ = 15.02, *p* < .001, *d* = 1.89).

This study was part of a larger study investigating friendships, romantic relationships, and sexuality between autistic males and females. Participants were recruited via different ways of recruitment in order to increase the possibility of recruiting a representative sample of the population. These methods included a series of paid online advertisements, contact with national and international autism awareness organisations, and support forums. In order to recruit a large autistic sample, additional sampling methods, including advertisements via social media outlets, word of mouth, and snowballing techniques were also used. All advertisements used language inviting autistic and non-autistic adults irrespective of birth sex to participate. Recruitment scripts also noted that the study aimed to investigate the social, romantic, and sexual experiences and relationships of participants. All methods of data collection were conducted in electronic format.

### Materials

A single internet-based questionnaire was used to collect data for this study. The questionnaire comprised demographic questions pertaining to participants’ age, sex assigned at birth, gender identity, diagnostic status, cultural and/or religious backgrounds, and level of education. The questionnaire was also comprised of two self-report measures: a diagnostic screening tool and a measure of sexual and romantic functioning (The Sexual Behaviour Scale, Version 3 [SBS-III] [51];). The SBS-III is a 236-item self-administered measure that examines reported levels of knowledge, attitudes, and behaviours across a range of social, sexual, and romantic domains. The measure is comprised of 14 subscales; however, one item examining sexual orientation (drawn from the Sexual Orientation subscale) and three items examining negative sexual experiences (drawn from the Sexual Behaviour subscale) were used in this study. The data examined in this study is drawn from the same dataset used by Pecora et al. [28]. Pecora et al. [28] examined negative sexual experiences across autism diagnosis and biological sex (males and females). As the rates of negative sexual experiences among females irrespective of gender identity and sexual orientation are reported in Pecora et al. [28], this study reports rates of negative sexual experiences among the same female sample, yet across gender identity and sexual orientation only.

#### Birth sex

The biological sex, or sex assigned at birth of participants was measured by responses to the single demographic item ‘at birth, I was considered’. This item contained the three response options of ‘male’, ‘female’, and ‘intersex’.

#### Age

Age was assessed by responses to the single demographic item ‘Your age is’. The age of participants was confirmed by assessing participant responses to the second demographic item assessing age, which required participants to report their date of birth.

#### Gender identity

Gender identity was assessed by responses to the single demographic item ‘I identify as’. This item contained the three response options of ‘male’, ‘female’, and ‘other’.

#### Sexual orientation

Sexual Orientation was examined via responses to the SBS-III item ‘I consider my sexual orientation to be’, which contained the six response options of ‘heterosexual’, ‘homosexual’, ‘bisexual’, ‘asexual’, ‘transattracted’, and ‘questioning’.

#### Autism diagnosis: the Autism Spectrum Quotient (AQ)—adult version

The AQ is a 50-item self-report questionnaire used to measure the degree to which autism-related traits are present in individuals (16 years or over). It contains five subscales of 10 items that assess the presence of deficits in (1) social skills, (2) attention switching, (3) attention to detail, (4) communication, and (5) imagination. All items are rated on a 4-point Likert scale ranging from 1 (definitely agree) to 4 (definitely disagree). Scores on all subscales are summed to create a total score (0–50), with higher scores indicating greater evidence of autism symptomatology. The authors of the AQ suggest a screening cut-off score of, or above 32, capturing 80% of individuals with autism at a 2% false-positive identification rate (sensitivity = .95, specificity = .52). Studies evaluating the psychometric properties of the measure support its reliability for use in Australian [52] and international samples [53, 54].

In the total sample, Cronbach's alpha for the AQ was 0.936. The mean AQ score for the autistic group was consistent with average scores identified in other autistic samples [50]. However, the mean AQ score, and large standard deviation within the non-autistic group was considerably higher than has been reported in non-autistic samples previously [50]. As analyses excluding and including non-autistic participants with higher AQ scores (> 17) showed little variation, the non-autistic group was fully retained to reduce bias, and maintain the benefits of a larger sample size.

#### Negative sexual experiences

For the purpose of this study, definitions of the negative experiences measured in this study are reported below:

- *Regretted sexual experiences*. Having consented to a sexual behaviour or experience that was regretted afterward. This is most commonly related to negative feelings that follow engagement in uncommitted sexual encounters (i.e. casual sex [45, 46]).
- *Unwanted sexual experiences*. Having consented to a sexual behaviour or experience that was unwanted at the time. This can include experiences of sexual coercion, unwanted sexual contact, and engagement in any form of sexual behaviour, including intercourse [47].
- *Unwanted advances from others*. Experiencing or being subject to advances from others that were unwanted. This can include unwelcome or unsolicited sexual behaviours, requests for sexual favours from others, and unwanted sexual attention [48].

Negative sexual experiences were assessed through responses to three items within the SBS-III: regretted sexual experiences ‘I have agreed to have sex with someone and regretted it afterward’, unwanted sexual experiences ‘I have agreed to have sex with someone that I didn’t want to’, and experiences of unwanted sexual advances ‘I have been the victim of unwanted sexual advances of behaviours from others’. Response options were ‘yes’ or ‘no’.

### Procedure

Ethical approval for the research was obtained from the governing institutional ethics committee. All procedures were in accord with the ethical standards defined in the 1964 Helsinki Declaration and its amendments. All participants provided fully informed consent. After consenting to participate in the survey, participants then completed the online questionnaire. The questionnaire was untimed and expected to take 30–45 min to complete.

### Data analysis

Data were screened for missing values, outliers, and multicollinearity for each participant group independently. Following the exclusion of two autistic cases with missing data on all subscales within the SBS-III, a total of 295 participants were retained. No outliers were identified in the dataset (*Z* > ± 3.29 [55]). Assessment for multicollinearity found all correlations between items to be below .90, and thus suggested independence of all models in the data [56]. As normality of variables is not required for samples with over 30–40 cases [57], the sample size was sufficient to render potential violations of normality as unproblematic, and as non-parametric procedures, such as the binomial logistic regressions (LRs) used in the current study, do not have distributional assumptions [55], issues of normality were not considered as a cause for concern. Examination of standardised residuals of all IVs and DVs (*p* > .05) indicated that the data met the assumption of linearity.

To determine if a greater proportion of autistic females compared to non-autistic females identified with transgender gender identities or non-heterosexual sexual orientations, frequency analyses were conducted via a series of proportion tests to compare the percentage of participants in each gender identity and sexual orientation sub-group. All proportion tests undertaken in this study utilised Stokes’ [58] Calculator-effect sizes, version 5.02.01. Following this, one binary LR was undertaken to compare reported (1) gender identity and (2) sexual orientation between autistic and non-autistic participants, in addition to the potential interaction between gender identify and sexual orientation. For this analysis, the independent variables (IV) were the range of gender identities (male, female, other) and sexual orientations that participants identified with. As there were no responses for an ‘asexual’, ‘transattracted’, or ‘questioning’ sexual orientation among participants in the sample, only the sexual orientations of ‘heterosexual’, ‘homosexual’, and ‘bisexual’ were examined in this study. Gender identity was included as an IV in step one, and both gender identity and sexual orientation were included as IVs in step two. The interaction between each IV (gender identity and sexual orientation) was included as step three of the LR. For this analysis, gender identity was dummy coded, with female gender identity used as the reference category. Sexual orientation was dummy coded as a categorical variable. ‘Heterosexual’ sexual orientation was used as the reference category. The dependent variable (DV) was diagnostic status (autism or no autism).

Six additional LR analyses were undertaken to determine if autistic and non-autistic individuals identifying as transgender or, with a non-heterosexual sexual orientation, were more likely to report each of the three negative sexual experiences than diagnosis-matched females reporting a female gender identity, or heterosexual sexual orientation. Three of the six LRs examined the autistic sample only. For these analyses, the IV was either the reported gender identity or sexual orientation of participants, which were entered in step one of the analysis. The remaining three analyses compared negative sexual experiences between autistic and non-autistic participants. For these analyses, diagnostic status was included as a second IV, in addition to gender identity and sexual orientation in step one. The interaction between gender identity and sexual orientation was included in step two of these analyses. The interaction between gender identity, sexual orientation, and diagnostic status (autism or no autism) was included in step three of these analyses. Participants who reported a ‘male’ and ‘other’ gender identity were combined into the single comparison group of ‘transgender’ gender identity. Sexual orientation was coded as a categorical variable, with heterosexual sexual orientation included as the reference category. For these six LRs, the three DVs were the reported frequency of ‘yes’ or ‘no’ responses to the three negative sexual experience items (regretted sex, unwanted sex, unwanted sexual advances).

For comparison purposes, three additional binary LRs were conducted examining negative experiences across gender identity and sexual orientation in the non-autistic sample. For these analyses, the IVs of gender identity and sexual orientation were entered in step one. The interaction between gender identity and sexual orientation was included as step two of the analysis. The ‘male’ and ‘other’ gender identity groups were combined into the single comparison group of ‘transgender’ gender identity. Sexual orientation was coded as a categorical variable, with ‘heterosexual’ sexual orientation used as the reference category. The three DVs were the reported frequency of ‘yes’ or ‘no’ responses to the three negative sexual experience items (regretted sex, unwanted sex, unwanted sexual advances).

Significant age differences were observed in this sample (*t*_(172.9)=_4.59 *p <* .001; *d =* 0.70), where the mean age of autistic females (*M =* 26.2 years, SD *=* 8.7, range 18–56 years) was higher than in the non-autistic group (*M =* 22.0 years, SD *=* 4.6, range 18–48 years). As similar recruitment methods were used for both the autistic and non-autistic sample and that no other demographic feature we collected discriminated between the groups, the cause of the significant age differences remains unclear. However, given that autistic individuals are often older when acquiring life skills [59], age was covaried for in all analyses in this study. Proportion tests comparing rates of comorbid diagnoses between the autistic and non-autistic sample also identified significant group differences in proportions of ADHD, depressive disorders, and ‘no/none’ reported comorbidities between the groups. Age, ADHD status, depressive disorder status, and ‘no/none’ reported comorbidities were controlled for in all LRs in this study. These variables were included in step three of these analyses. All non-significant results of control variables were not reported in the results.

## Results

### Demographic trends

Comparisons between the percentages of autistic and non-autistic females who identified as transgender and with a non-heterosexual sexual orientation were undertaken using proportion tests. These results are summarised in Table 2.

**Table 2:** Proportions of autistic and non-autistic females on gender identity and sexual orientation

		Autistic (*n* = 134)	Non-autistic (*n* = 161)	*Z*_Diff_	*p* value
		*n* (%)	*n* (%)		
Gender identity	% Female	108 (80.6)	147 (91.3)	2.50	.003
	% Male	4 (3.0)	2 (1.2)	1.25	.105
	% Other	22 (16.4)	12 (7.5)	2.45	.032
Sexual orientation	% Heterosexual	41 (30.6)	88 (54.6)	3.30	< .001
	% Bisexual	34 (25.4)	27 (16.8)	1.70	.048
	% Homosexual	59 (44.0)	46 (28.6)	2.68	.003
	% Asexual	0 (0)	0 (0)	n/a	n/a
	% Transattracted	0 (0)	0 (0)	n/a	n/a
	% Questioning	0 (0)	0 (0)	n/a	n/a

Sexual orientation of participants has been drawn from the same dataset as Pecora et al. [28]. Proportions of reported sexual orientation by diagnostic group and birth sex have been reported in Pecora et al. [28]

### Gender identity and sexual orientation

Given the limited sample reporting a male gender identity (autistic *n* = 4; non-autistic *n* = 2) or other gender identity (autistic *n* = 22; non-autistic *n* = 12), these two groups were collapsed to create separate autistic and non-autistic groups identifying as transgender (male or other).

LR examining gender identity and sexual orientation in the autistic versus non-autistic sample identified that a diagnosis of autism significantly predicted gender identity and sexual orientation (*R*^2^_LL_ = 0 .04, *χ*^2^_(4)_ = 18.29 *p* < .001). Autistic females were 1.68 (*p* = .166) times as likely to report a transgender gender identity than non-autistic females. These results should be considered in the context of the limited number of non-autistic transgender females in this study (*n* = 14) and interpreted with caution.

Examination of sexual orientation identified that autistic females were more likely to identify with a non-heterosexual sexual orientation than their non-autistic counterparts (*p* = .004). Results indicated that, compared to females without autism, autistic females were 2.39 (*p* = .007) times more likely to report a homosexual sexual orientation, and 2.33 (*p* = .003) times more likely to report a bisexual sexual orientation (Table 3). The interaction between gender identity and sexual orientation was not significant (*R*^2^_LL_ = 0 .05, *χ*^2^_(3)_ = 20.54, *p* = .753).

**Table 3:** LR comparing transgender gender identity to a ‘female’ gender identity, and non-heterosexual sexual orientations to a ‘heterosexual’ sexual orientation between autistic and non-autistic females

IV					
Gender identity	B	SE	OR	95% CI	*p*
Transgender	0.52	0.38	1.68	0.81–3.51	.166
**Sexual orientation**	**B**	**SE**	**OR**	**95% CI**	***p***
Homosexual	0.87	0.33	2.39	1.26–4.52	.007
Bisexual	0.85	0.29	2.33	1.33–4.09	.003
**Gender identity**-sexual orientation*	**B**	**SE**	**OR**	**95% CI**	***p***
	0.26	0.71	1.29	0.32–5.18	.718

Note: Reference category (IV1) = female gender identity. Reference category (IV2) = heterosexual sexual orientation. (DV) = autism diagnosis.. 95% CI is reported for the OR. Age and the comorbid diagnoses of ADHD status, major depression, and ‘no’ reported comorbid diagnoses were controlled for in all analyses. As each controlling covariate was non-significant in all analyses, we do not present or interpret these results

*Interaction between IVs

### Gender identity and negative sexual experiences

Autistic transgender females were compared to autistic cisgender females to establish if a transgender gender identity increased the likelihood of reporting negative sexual experiences. Group differences based on reported gender identity within the autistic group were not significant across all DVs (*p* ≥ .63).

Results for a transgender gender identity (within autism) were then compared to a transgender identity without autism. No significant differences were found between autistic and non-autistic participants identifying with a transgender gender identity over all DVs (*p* ≥ .08).

As a comparison to the autistic respondents, negative sexual experiences were examined within the non-autistic sample alone (Table 4). Non-autistic transgender females were 4.01 times more likely to report engaging in a regretted sexual experience than non-autistic cisgender females (*p* = .024). No differences were found between groups for unwanted sexual behaviours, and experiences of unwanted sexual advances.

**Table 4:** Summary of significant LR results comparing negative sexual experiences across gender identity and sexual orientation

Comparison (IV groups)	DV	*R*^2^_LL_	*χ*^2^_(3)_	B (SE)	OR	95% CI	*P,* Wald test
**Transgender GI (non-Aut) versus cisgender GI (non-Aut)**	Regret. behaviour	0.03	5.72	1.39 (0.62)	4.01	1.20–13.42	.024
	Interaction (non-Aut versus non-Aut comparisons)						≥ .159
**Homosexual SO (Aut) versus heterosexual SO (Aut)**	Regret. behaviour	0.04	5.88	1.01 (0.42)	2.72	1.19–6.20	.017
	Unwant. behaviour	0.06	7.60	1.16 (0.43)	3.17	1.36–7.38	.007
	Interaction (Aut versus Aut comparisons)						≥ .588
**Homosexual SO (Aut) versus heterosexual SO (non-Aut)**	Unwant. behaviour	0.06	12.95	1.09 (0.39)	2.97	1.38–6.42	.005
	Unwant. advances	0.04	17.09	1.08 (0.36)	2.94	1.46–5.90	.002
	Interaction (Aut versus non-Aut comparisons)						≥ .318
**Homosexual SO (non-Aut) versus heterosexual SO (non-Aut)**	Unwant. advances	0.04	6.89	0.98 (0.38)	2.67	1.26–5.63	.010
**Homosexual SO (Aut) versus homosexual SO (non-Aut)**	Unwant. behaviour	0.03	4.69	0.87 (0.41)	2.38	1.07–5.22	.033
**Bisexual SO (Aut) versus heterosexual SO (non-Aut)**	Regret. behaviour	0.03	4.89	− 0.97 (0.46)	0.38	0.15–0.93	.034

Note: Only significant results are reported in Table 4. *Regret. behaviour* having engaged in sexual behaviours that were regretted, *Unwant. behaviour* having engaged in unwanted sexual behaviours, *Unwant. advances* having received or been the victim of an unwanted sexual advance, *Aut* autistic, *non-Aut* non-autistic controls, *GI* gender identity, *SO* sexual orientation, *DV* reported negative sexual experience (regretted, unwanted sexual experience or unwanted sexual advance). Reference category = ‘female’ for all analyses investigating gender identity. Reference category = ‘heterosexual’ for analyses investigating sexual orientation. Age and the comorbid diagnoses of ADHD status, major depression, and ‘no’ reported comorbid diagnoses were controlled for in all analyses. As each controlling covariate was non-significant in all analyses, we do not present or interpret these results

### Sexual orientation and negative sexual experiences

Negative sexual experiences were examined across sexual orientation. Autistic females identifying as homosexual were 2.72 (*p* = .017) times more likely to have experienced a regretted sexual behaviour and 3.17 (*p* = .007) times more likely to have experienced an unwanted sexual behaviour compared to autistic females identifying as heterosexual (Table 4)*.* Differences in rates of unwanted advances from others between autistic homosexual and heterosexual females were not significant. No significant differences were found between bisexual and autistic heterosexual females on any of the dependent variables.

Negative sexual experiences were examined between autistic and non-autistic females (see Table 4). Autistic homosexual females were more likely to report an unwanted sexual experience (OR = 2.98; *p* = .005), and unwanted advance (OR = 2.94; *p* = .002) than non-autistic heterosexual females. Autistic homosexual females were also more likely to report an unwanted sexual experience than non-autistic homosexual females (OR = 2.38; *p* = .033). No differences were found for rates of regretted sexual experiences between autistic homosexual females and non-autistic females with both a heterosexual and homosexual orientation. Differences in experiences of unwanted sexual advances between autistic homosexual females and non-autistic homosexual females were likewise, not significant.

Autistic bisexual females were compared to non-autistic heterosexual females. Autistic bisexual females were less likely to report having engaged in a regretted sexual behaviour (OR = 0.38; *p* = .034) than non-autistic heterosexual females. No other differences were found between autistic bisexual and non-autistic heterosexual females. The likelihood of reporting any of the three negative sexual experiences was similar for bisexual females with and without autism.

The non-autistic sample was examined for purposes of comparison (Table 4). Analyses revealed that compared to non-autistic females identifying as heterosexual, non-autistic homosexual females were 2.67 (*p* = .010) times more likely to have experienced an unwanted sexual advance. There were no differences between non-autistic heterosexual and homosexual females in their reported engagement in unwanted sexual behaviours, or unwanted advances. Differences between heterosexual and bisexually orientated non-autistic females across all negative experiences were likewise, not significant. The interaction between gender identity and sexual orientation in all analyses within this study were not significant (*p* ≥ .159; Table 4).

## Discussion

The aim of the current study was to explore the representation of transgender gender identities or non-heterosexual sexual orientations in autistic females, in addition to the prevalence of negative sexual experiences among autistic females within these minority groups. As hypothesised, autistic females were more likely to identify with both a transgender gender identity, and non-heterosexual sexual orientation than non-autistic peers. Frequency analyses also identified that a greater proportion of autistic females reported having experienced unwanted sex and sexual advances compared to non-autistic females.

Contrary to expectations, identifying as transgender did not appear to influence the likelihood of having a negative experience in autistic individuals. However, non-autistic transgender individuals were more likely to report a regretted sexual experience than cisgender non-autistic participants. This finding should be interpreted in the context of the small numbers of participants identifying as transgender within both autistic and non-autistic samples.

The hypothesis that autistic non-heterosexual females would be more likely to report a negative sexual experience than autistic heterosexual females and non-autistic females irrespective of sexual orientation was partially supported. Autistic homosexual females had an increased risk of (i) unwanted sexual behaviour compared to heterosexual females with and without autism and (ii) regretted sexual behaviours compared to autistic heterosexual females. Surprisingly, autistic bisexual females had a reduced risk of regretted sexual behaviour compared to non-autistic heterosexual females. No differences were found in all remaining comparisons between autistic females identifying with a non-heterosexual sexual orientation and non-autistic peers. For non-autistic females, we found that identifying as homosexual increased the risk of unwanted advances, but not of unwanted or regretted sex compared to non-autistic heterosexual females. Bisexuality also provided a protective effect in females without autism, reducing risk of regretted sexual experiences but not of unwanted sexual experiences or advances, compared to non-autistic heterosexual females. These findings support the hypotheses predicting an increased risk of negative sexual experiences among autistic homosexual females, yet not for transgender females, and bisexual females. However, findings pertaining to negative sexual experiences across sexual orientation should be interpreted in the context of the increased proportion of participants identifying as homosexual (45.6%) in the non-autistic group.

The results of this study contribute to emerging insights of a higher proportion of transgender gender identities among autistic female populations compared to non-autistic populations [2, 22] and support proposed relationships between autism and GD [3, 5, 22]. Study findings also provide further evidence of an increased sexual diversity (i.e. higher incidence of homosexual or bisexual orientation, and lower incidence of heterosexual orientation) in autistic female groups, compared to the general population. This is consistent with existing research [18, 20–22].

Various hypotheses have been proposed to explain the increased diversity in both gender identity and sexual orientation among autistic females. These include a prominent neurobiological perspective, which suggests that an overexposure to foetal testosterone may lead to an increased development of male-based traits, and a preference towards masculinised gendered behaviours and activities within females [60]. Foetal exposure to testosterone affects aspects of adult personality including a male-oriented gender identity [61] and sexual orientation within the broader population [62]. As evidence of elevated testosterone has been found in autistic females [63, 64], greater exposure to foetal testosterone has been conceptually linked to increased likeliness of developing a male gender identity, a sexual preference towards females, and thus a homosexual and/or bisexual sexual orientation among autistic females [64].

Although foetal testosterone theory would account for variation in gender identity and sexual orientation in females, autistic males also present with more diverse sexual identities than non-autistic males [21, 65]. There is some weak evidence that suggests that increased exposure to foetal testosterone may be implicated in the development of a homosexual orientation in cisgender males [66, 67]. Other studies have also suggested excessively high foetal testosterone may predispose males to develop a homosexual sexual orientation [68], and feminise autistic males [69]. Despite these findings, conclusions drawn from two more recent reviews of current data examining prenatal influences on sexual orientation suggest that there are no meaningful differences in the level of exposure to foetal testosterone between heterosexual and homosexual males [70, 71]. Rather, the evidence suggests that the differences in sexual orientation among males are due to the variation in each individual’s response to prenatal testosterone, rather than level of exposure to it [70]. Interestingly, the review also suggests that the variation in exposure to prenatal testosterone is more consistently associated with sexual orientation in females rather than males [70, 72]. Considering these inconsistencies, it is likely that different prenatal and biological mechanisms play a role in the development of sexual orientation for both males and females [72]. Thus, further research is required to decipher the links between foetal testosterone and sexual identity between autistic and non-autistic individuals of both sexes.

Other explanations are consistent with social motivation theories [73, 74]. For example, features of autism include rigid thought processes and obsessional interests, and these may lead to inflexible interpretations of gender roles and increased likelihood of developing a gender identity that is not consistent with one’s birth sex if interests/attributes do not fit with stereotypes [5, 11]. Other features of autism, including lower sensitivity to social norms [74] together with reduced access to sexual and romantic partners experienced by many autistic people [22, 25], have also been proposed to reduce the relevance of birth sex when choosing or responding to potential partners [75], and increase the fluidity of sexual preferences and practices [14]. The complexities surrounding the relationship between autism and variations in gender identity and sexual orientation are likely to involve a number of interacting mechanisms rather than a single factor, and so multivariate hypotheses [6] may provide a more accurate explanation for our observations.

In addition to the increased variation in gender identity and sexual orientation within autism, insight into the nature of sexual experiences of transgender individuals is an important contribution of this study. This study found that identifying with a transgender identity increased the risk of negative sexual experiences, specifically, regretted sexual experiences for non-autistic individuals only. To date, research is yet to investigate the nature and circumstance of regretted and unwanted sexual behaviours across gender identity in either autistic or non-autistic samples. However, the finding of an increased risk of regretted sexual experience among non-autistic transgender individuals partially aligns with research showing increased risks for sexual victimisation [76] and intimate partner violence [34] among transgender females in the broader population. As the non-significant differences between transgender and cisgender autistic individuals across all negative sexual experiences in this study contrast against this research, clarification of the nature of sexual victimisation among minority and mainstream populations identifying with transgender gender identities should be a priority for future research.

The patterns of findings across the different negative sexual experiences measured provide some information about the nature of adverse sexual experiences of individuals with and without autism, and about how sexual orientation is an additional risk and protective factor. Our findings show that a homosexual sexual orientation is linked to increased risk of having experienced both regretted and unwanted sexual behaviour, but not unwanted advances, in autistic females, and an increased risk of unwanted sexual advance in non-autistic females. We also found that autistic homosexual females present with an increased risk of experiencing unwanted sexual behaviours than non-autistic heterosexual females, and are at an increased risk of unwanted advances than both non-autistic homosexual and heterosexual females. Bisexual females irrespective of diagnosis are at a lower risk of a regretted sexual behaviour than non-autistic heterosexual peers. These results align with findings of broader literature reporting higher prevalence of sexual victimisation between homosexual females than the general population [32, 77]. They likewise agree with research involving female-only samples, in which sexual victimisation has been found to be twice as likely between homosexual, compared to heterosexual women (e.g. 66% vs 38% respectively [78, 79]).

The results contrast with research investigating sexual victimisation in bisexual females in the broader population, where bisexual females have reported a higher rate of sexual victimisation [78]. Specifically, bisexual females reported a higher lifetime prevalence of sexual violence and victimisation (74.9–78%) than heterosexual (38–43.3%) and homosexual females (46.4–66% [78, 79]). Bisexual females have also been found to be up to 1.9 and 2.6 times more likely to have experienced intimate partner violence than homosexual and heterosexual peers, respectively [80], and have experienced more frequent and severe cases of sexual victimisation and abuse than females identifying with a heterosexual or homosexual sexual orientation [78, 81]. The reason for the differences in rates of negative sexual experiences between homosexual and bisexual females within this study and inconsistencies with previous research remain unclear. However, the most commonly identified risk factors for sexual victimisation among bisexual females include an increased prevalence of risky alcohol use [79], and a greater number of lifetime sexual partners, which increase the likelihood of exposure to potential sexual aggressors [78, 79]. Given that autistic individuals report less sexual experience [27, 82] and fewer sexual partners [27] than non-autistic counterparts, it is possible that the bisexual autistic participants in this study may have had less exposure to some of these risk factors than the bisexual females in previous research. Moreover, as homosexual females in the general population are more likely to report negative sexual consequences related to alcohol consumption [83] and are more likely to meet the diagnostic criteria for substance use (see [49]) than bisexual females [83], it is also possible that risky alcohol consumption may have also been a factor that played a role in some of the negative experiences reported by homosexual females in this study. Finally, as this study examined constructs of regretted and unwanted sexual experiences and advances, yet previous research has investigated rates of sexual victimisation [79], sexual abuse [78], and intimate partner violence [80] across sexual orientation, it is also possible that inconsistencies with previous research may be partially due to the differences in the specific variables measured across studies. Despite this, research is still required to determine the factors that increase risks to negative sexual experiences, and the contexts to which these occur for non-heterosexual females with and without autism.

The increased risk of negative sexual experiences between homosexual females with and without autism found here is concerning. Research is yet to explore the nature of adverse sexual experiences across sexual orientation within autism. In the general population, literature examining these variables is limited to prevalence data [78, 84] that do not offer explanations for the increased rates of sexual victimisation among homosexual females. The studies do highlight that cases of sexual victimisation (81% [78]) and unwanted sexual contact (85.2% [84]) among homosexual females often involve male perpetrators. Moreover, homosexual females also report aversive experiences with the opposite sex at younger ages and more negative attitudes towards previous sexual interactions with males than heterosexual counterparts [85]. Although still in need of empirical validation, Harrison et al. [85] have considered the possibility that the increased rates of unwanted sexual experiences among homosexual females may have occurred with those of the opposite sex during critical periods of sexual identity development. Thus, it is possible that the regretted or unwanted sexual experiences cited among homosexual females in this study may have occurred with individuals of the opposite sex before a clear sexual identity was developed. However, research that aims to determine if negative sexual experiences, or a vulnerability to unwanted sexual events, may shape the development of an individuals’ sexual orientation development in autistic and non-autistic circles is still necessary.

### Limitations

The results of this study should be interpreted in light of their limitations, which carry important implications for future research. As this was a quantitative study, participant responses were based on a series of predetermined test items and response options. As such, response options that allowed for participants to qualitatively expand on their reported gender identities or sexual orientations were not available when completing the survey. If participants identified with a specific gender identity (i.e. non-binary, transmale) or sexual orientation (i.e. demisexual, pansexual) that was not included as a response option, this may have prevented participants from selecting or expanding on the specific sexual identity that they identified with.

Similarly, the design of test responses also limited abilities to expand on the nature, conditions, or frequency of each negative experience. Thus, findings cannot provide insight into the reasons why, and under what conditions these experiences occurred, as well as the factors that led participants to view these experiences as regretted or unwanted. This also limits the extent to which age-related patterns could be explored and how the development of a clear sexual identity or long-term relationships may shape perceptions of previous sexual interactions with former partners. Such data would provide valuable information that could facilitate a more sensitive comparison between autistic and non-autistic females of different gender identities and sexual orientations. Findings would also inform interventions to help females of minority groups avoid and manage the outcomes of negative sexual experiences.

Secondly, while sexual orientation was measured via participants’ self-reported sexual identity, sexual orientation also encompasses an individual’s sexual attraction and contact with others [14]. Thus, this study did not consider participants’ sexual attractions or behaviours when distinguishing between sexual orientation labels. As there can be some inconsistency between an individuals’ sexual identity, attractions and behaviours [86], participants’ sexual identity may or may not truly align with their attractions and behavioural experiences. Thus, while relying on participants’ self-reported sexual identity may be related to an individual’s attractions and behaviours, measuring all three domains of identity, attraction, and behaviours would be more informative.

Given the limited number of autistic (*n* = 26) and non-autistic (*n* = 14) transgender females in this study, the comparisons made using smaller participant subgroups may be limited by poor power. Consequently, our attempt to draw conclusions about the links between an individuals’ gender identity and their risks to adverse sexual experiences in both the autistic and non-autistic groups is tentative. Although the data in this study should be interpreted cautiously, findings highlight the importance for future research to clarify the role of gender identity in risks and rates of negative sexual experiences among females irrespective of diagnosis.

A large proportion of participants in the non-autistic group (45.6%) reported a non-heterosexual sexual orientation. This figure is substantially higher than that observed in the broader population, which ranges from 2 to 5% [87, 88]. Although participants were recruited via a range of various online platforms, the use of social media advertisements and snowballing techniques, which were used to recruit autistic participants, do not completely ensure that a representative sample was attained. As it is possible that the sample is not entirely representative of the broader population, the rates of sexual orientation in this study should be interpreted with caution. Moreover, it is also possible that some may have had a pre-existing interest in sexuality when volunteering to participate in this study, the overrepresentation of non-heterosexuality in this group may have been due to a bias in participant selection. Despite this, the increased rates of a non-heterosexual orientation between autistic females when compared to non-autistic females in this study may have been even more pronounced if the prevalence of non-heterosexuality within the non-autistic group (45.6%) reflected estimates observed within the wider population (2–5%).

As this study examined patterns of sexuality and sexual victimisation among autistic females, study conclusions cannot be generalised to the broader autistic population irrespective of sex. Given that transgender and gay males experience forms of sexual violence and coercive behaviours [77], the increased rates of adverse sexual experiences in this study cannot be fully attributed to an autism diagnosis or female birth sex. It would therefore be valuable to examine whether rates of negative sexual experiences are also elevated among autistic males who identify as transgender, or with a non-heterosexual orientation when compared to non-autistic male counterparts. These findings would clarify whether increased sexual risks are unique to autistic females who present with diverse sexual identities, or if these vulnerabilities are apparent among the autistic transgender or autistic non-heterosexual population overall.

Finally, this study did not test for dependence between the dependent variables of each negative sexual experience. As such, whether participants who endorsed a regretted, an unwanted sexual event, or an unwanted sexual advance would be more likely to also report another negative sexual experience was not examined in this study. This may bias the results, increasing the likelihood of us finding a result where a regretted event was reported because of an unwanted event.

### Future directions

The findings of this study assert the importance of continuing to further understand the sexuality of autistic females, and the factors that shape their sexual identities and sexual experiences. Further research is required to continue investigating the links between sexual diversity and autism, as well as the mechanisms underlying the development of identity and attraction for autistic females. Although this study identified an increased prevalence of negative sexual experiences among autistic, homosexual females, the reasons behind these elevated rates remain unclear. Accordingly, research that explores the nature and characteristics of regretted and unwanted sexual experiences between autistic females across sexual orientation may identify the causal mechanisms leading to these increased sexual vulnerabilities.

Additionally, this study did measure the construct of regretted sexual experiences using definitions from prior literature that conceptualised such experiences as the (consensual) engagement in sexual behaviours that are later associated with negative emotions [45, 46]. However, the potential reasons that sexual behaviours were viewed and reported as regretted by participants were not examined in this study. While there are a number of reasons why a sexual behaviour could be viewed as regretted [89], it is possible that some of these experiences are due to an individual not knowing what to expect when initially consenting to engage in a (later regretted) sexual behaviour [89]. Other times, sexual interactions can be spontaneous, evolving so quickly that an individual may only realise that they did not want, or enjoy the experience until after it had ended [89, 90]. As such, future research would benefit from exploring the emotion of regret in relation to past sexual behaviours, as well as the factors that have led each individual to perceive a particular experience as regretted. Moreover, as regretted experiences have been associated with poor psychological health and wellbeing [90] and may also influence both future sexual behaviours and choices of sexual partners [89], exploring the links between these variables in both autistic and non-autistic samples is necessary.

Finally, although evidence of poor mental health outcomes has been observed among individuals who identify with a sexual minority that have experienced sexual victimisation within the broader population [39], they are yet to be examined within autism. Thus, further research is required to determine the specific mental health challenges that are and are not unique to autistic females who identify with a sexual minority, and have also been subject to a negative sexual experience. These findings may identify the extent to which negative sexual experiences may be amplifying the mental health difficulties already apparent for autistic individuals [41, 42], while also identifying the most effective practices to prevent these outcomes.

### Clinical implications

In light of the sexual vulnerabilities that are already apparent for autistic females, the additional risks identified in this study signal the importance of addressing the needs of autistic females with a homosexual sexual orientation. The results of this study therefore hold practical and clinical implications for health care providers supporting both autistic females and those that identify with a sexual minority. It is apparent that there is a need to increase clinical awareness of the higher representation of gender and sexual diversity in autism, particularly among autistic females. Clinicians should be prepared to provide opportunities to share the concerns and uncertainties these individuals may be experiencing as they develop and express their sexual and gender identities. Professionals should likewise be aware of the unique challenges and health care needs that autistic females may experience when identifying within a gender or sexual minority. These include poorer levels of psychological wellbeing and higher rates of internalising problems [26, 91], which are also compounded by increased risks of complex mental health issues and socioemotional problems that individuals identifying with a sexual minority are often subject to [37, 38, 92]. Through increased understanding, this knowledge can also be used to develop strategies and services that aim to provide these females with the skills required to develop a clear and fulfilling gender and sexual identity.

The findings of increased rates of regretted and unwanted sexual experiences among autistic homosexual females raise a number of immediate concerns. Results highlight the importance of increasing professional awareness of the increased risks to adverse sexual experiences among individuals identifying within a multiple minority group. Findings assert the need for clinicians to understand the specific types of negative sexual experiences that autistic and non-autistic females are at risk of experiencing, and the role that sexual orientation plays in increasing or reducing this level of risk. Given that irrespective of diagnosis, homosexual females were more likely to have engaged in unwanted sexual behaviours, and autistic homosexual females also cited increased regretted experiences, findings highlight the importance of clinicians to assess the sexual history of autistic, and homosexual, females in sensitive ways. Thus, it would be helpful to explore the extent to which engagement in unwanted behaviours may influence feelings of regret with clients and address reasons why individuals may be engaging in unwanted and regretted sexual behaviours. Moreover, as homosexual females with and without autism also present with increased risks of unwanted sexual advances than non-autistic heterosexual peers, clinicians could also work with clients to explore their feelings around these experiences and develop personal boundaries to protect their safety.

Professionals should likewise be attuned to recognise the risk factors, warning signs, and symptoms of sexual victimisation, and previous unwanted sexual experiences within their clients. Tailored support programmes would benefit from greater understanding of multiple levels of risk, and unwanted sexual experiences that autistic, and sexual minority females may be subject to. These educational efforts could aim to promote the sexual health and safety of autistic, and non-heterosexual females, to enhance personal skills required to make safe and positive decisions in sexual situations. Thus, these programmes may be one of the first steps in reducing the sexual risks and vulnerabilities among autistic and non-autistic homosexual females and may also allow these females to cope with the adverse psychological and interpersonal outcomes that often accompany aversive negative events.

## Conclusions

The results of this study yielded some important findings. Results support and extend upon previous literature that suggests an increased diversity of gender identities and sexual orientations within autism compared to non-autistic populations [6, 92]. Being the first study to explore the nature of negative experiences in autistic and non-autistic females across gender identity and sexual orientation, our findings provide insight into the nature of sexual experiences particularly for females who identify as homosexual within these groups. Consequently, they have highlighted the elevated sexual risks and vulnerabilities that are apparent for autistic females who identify within multiple minority groups, and the impact this may have on their health and wellbeing. Despite this, an understanding of the nature and cause of these increased risks among females within these groups is still required. Thus, findings provide foundations for future research that examines the sexual experiences of autistic females across gender identity and sexual orientation, while also informing interventions to facilitate the reduction of these risks and vulnerabilities.

## Data Availability

The dataset used and analysed during the current study are available from the corresponding author on reasonable request

## Abbreviations

ASD: Autism spectrum disorder
AQ: Autism spectrum quotient
DV: Dependent variable
GD: Gender dysphoria
IV: Independent variable
LR: Binary logistic regression
SBS-III: Sexual Behaviour Scale (Version 3)
